# Theoretical study of the frequency shift in bimodal FM-AFM by fractional calculus

**DOI:** 10.3762/bjnano.3.22

**Published:** 2012-03-07

**Authors:** Elena T Herruzo, Ricardo Garcia

**Affiliations:** 1 IMM-Instituto de Microelectrónica de Madrid (CSIC). C Isaac Newton 8, 28760 Madrid, Spain

**Keywords:** AFM, atomic force microscopy, bimodal AFM, frequency shift, integral calculus applications

## Abstract

Bimodal atomic force microscopy is a force-microscopy method that requires the simultaneous excitation of two eigenmodes of the cantilever. This method enables the simultaneous recording of several material properties and, at the same time, it also increases the sensitivity of the microscope. Here we apply fractional calculus to express the frequency shift of the second eigenmode in terms of the fractional derivative of the interaction force. We show that this approximation is valid for situations in which the amplitude of the first mode is larger than the length of scale of the force, corresponding to the most common experimental case. We also show that this approximation is valid for very different types of tip–surface forces such as the Lennard-Jones and Derjaguin–Muller–Toporov forces.

## Introduction

Since the invention of the atomic force microscope (AFM) [1], numerous AFM studies have been pursued in order to extract information from the sample properties in a quantitative way [2–16]. Both static (contact) [2–7] and dynamic [8–11,14–17] AFM methods have been applied. Static techniques such as nanoindentation [2], pulsed-force mode [3] and force modulation [4–6] are able to extract quantitative properties of the sample in a straightforward manner, but they are usually slow and invasive. Although these techniques allow control of the force applied to the sample, they are usually limited to forces above 1 nN, and such forces can damage the structure of soft samples.

On the other hand, AFM techniques based on dynamic AFM modes have the ability to make fast and noninvasive measurements. They are potentially faster because the quantitative measurements can be acquired simultaneously with the topography. In addition, the lateral forces applied to the sample can be smaller, which minimizes the lateral displacement of the molecules by the tip. Moreover, dynamic modes have already demonstrated their ability to map compositional properties of the sample [11,18–19]. However, quantifying physical properties is hard, because a direct relationship between observables and forces is difficult to deduce.

Since the observable quantities in dynamic modes are averaged over many cycles of oscillation (amplitude and phase shift for amplitude modulation AFM (AM-AFM) [20–21], and frequency shift and dissipation for FM-AFM [22–23]), it is not straightforward to obtain an analytical relationship between observables and forces. It is known that in FM-AFM the frequency shift of the first mode can be directly related to the gradient of the force when the amplitude is much smaller than the typical length scale of the interaction. For larger amplitudes, the frequency shift is related to the virial of the force [24–25]. Sader and Jarvis have proposed an alternative interpretation of FM-AFM in terms of fractional calculus [26–27]. They showed that the frequency shift can be interpreted as a fractional differential operator, where the order of differentiation or integration is dictated by the difference between the amplitude of oscillation and the length scale of the interaction.

Successful approaches to reconstruct material properties in a quantitative way came along with the development of novel AFM techniques, such as scanning probe accelerometer microscopy (SPAM) [8,28], or by making use of higher harmonics of the oscillation in order to relate the force with the observable quantity through its transfer function [11]. In particular, the torsional-harmonic cantilevers introduced by Sahin et al. allowed the reconstruction of the effective elastic modulus of samples in air [14] and liquids [29–31].

**Figure 1:**
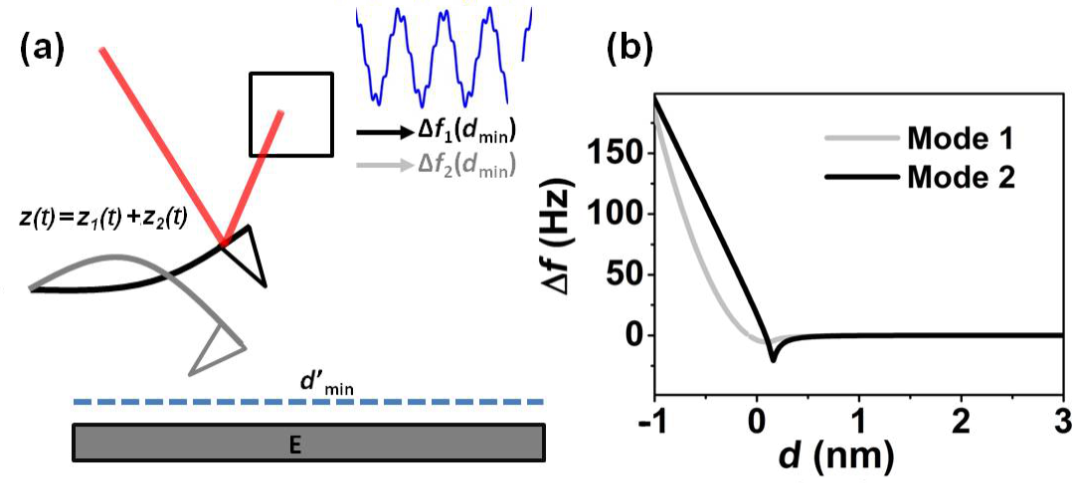
(a) Scheme of the first two eigenmodes of a cantilever and the tip deflection under bimodal excitation. In bimodal FM-AFM changes in the interaction force produce changes in the resonant frequency. The feedback loop keeps the resonant frequency of the 1*^st^* mode constant by changing the minimum tip–surface distance. (b) Frequency shifts of the 1*^st^* and 2*^nd^* modes as a function of the tip–surface distance.

Bimodal AFM [32–33] is a force-microscopy method that allows quantitative mapping of the sample properties (Figure 1). Bimodal AFM operates by exciting simultaneously the cantilever at its first and second flexural resonances. The technique provides an increase in the sensitivity toward force variations [15,18–19,33–36] with respect to conventional AFM. At the same time, it duplicates the number of information channels, through either the amplitude and phase shift of the second mode in bimodal AM-AFM, or the frequency shift Δ*f*_2_ and dissipation of the second mode in bimodal FM-AFM. Experimental measurements have shown the ability of bimodal AFM to measure a variety of interactions, from electrostatic to magnetic or mechanical, both in ultrahigh vacuum [36–38], air [33–34,39–41] and liquids [15,18–19]. Furthermore, it is compatible with both frequency-modulated [15,36–38] and amplitude-modulated AFM techniques [18–19,33–34,39–41]. Recently, Kawai et al. [36] and Aksoy and Atalar [42] found a relationship between Δ*f*_2_ and the average gradient of the force over one period of oscillation of the first mode.

Here, we propose a theoretical approach to determine the frequency shift in bimodal FM-AFM in terms of a fractional differential operator of the tip–surface interaction force. The frequency shift of the second mode is related to a quantity that is intermediate between the interaction force and the force gradient. This quantity is defined mathematically as the half-derivative of the interaction force. This approach does not make any assumptions on the force law, and it explains the advantages of bimodal FM-AFM with respect to conventional FM-AFM whenever the amplitudes of the first mode are larger that the characteristic length of scale of the interaction force.

## Results and Discussion

### Frequency shift of the second mode in bimodal AFM

The problem of a cantilever vibrating under bimodal excitation can be studied by means of the averaged quantities of the dissipated energy and the virial [43–45]. The virial of the *n^th^* mode is defined as

[1]
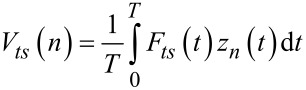


where *t* is the time and *T* is the period of the oscillation

The tip deflection in bimodal FM-AFM can be described as:

[2]
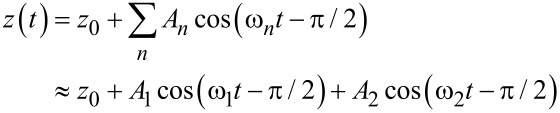


where *z*_0_ is the mean deflection, and *A_n_* and ω*_n_* are the amplitude and the frequency of the *n^th^* mode.

By substituting Equation 2 into Equation 1 and replacing *F_ts_* by its equivalent according to the Newton equation, an expression for the virial of the second mode that applies to bimodal FM-AFM is deduced [45]

[3]
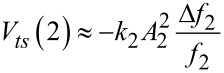


An additional approximation can be performed by considering that the free amplitude of the second mode *A*_2_ is much lower than the free amplitude of the first mode (*A*_2_ << *A*_1_) [15,36,42]. In this case *z*(*t*) can be expanded in powers of *A*_2_cos(ω_2_*t* − π/2), and the virial of the second mode is given by

[4]
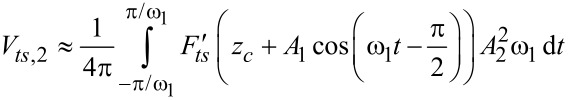


where *z_c_* is the average cantilever–sample separation.

By combining Equation 3 and Equation 4 we deduce a relationship between the second-mode parameters and the gradient of the force averaged over one cycle of the oscillation of the first mode.

[5]
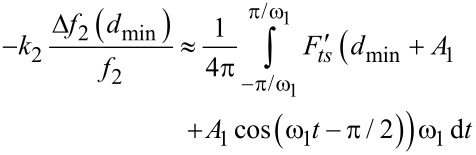


where *f_n_* = ω*_n_*/2π, and *d*_min_ is the minimum distance between tip and sample (*d*_min_ ≈ *z_c_* − *A*_1_).

### Interpretation of the frequency shift in bimodal FM-AFM in terms of the half-derivative of the force

By defining a new variable *u* = *A*_1_cos(ω*t* − π/2), the frequency shift of the second mode (Equation 5) can be expressed as the convolution of the force gradient with the function 
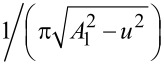
, in the same way that the frequency shift of the first mode in conventional FM-AFM can be seen as the convolution of the force gradient with the semicircle 
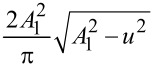
 [24]:

[6]
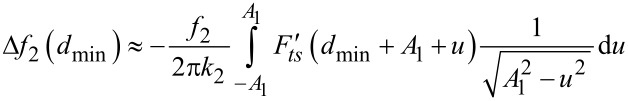


By using the definition of the Laplace transforms of the force *F*(*z*) and its derivative *F*^′^(*z*)

[7]
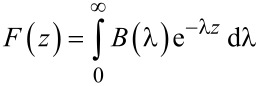


[8]
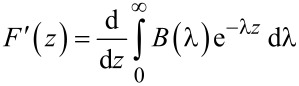


By substituting Equation 8 in Equation 6 we have

[9]
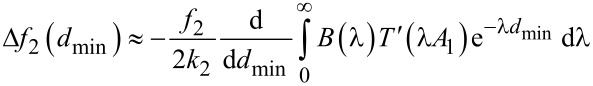


where

[10]
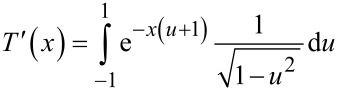


*T*^′^(*x*) can be expressed in terms of the modified Bessel function of the first kind of order zero *I*_0_(*x*) (*T*^′^(*x*) = *I*_0_(*x*)*e*^−^*^x^*) [46]. By comparing Equation 8 and Equation 9, it can be seen that Δ*f*_2_ is related to the gradient of the force through the derivative operator and a function *T*^′^(λ). By analogy with the Sader and Jarvis method to express the frequency shift of the first mode in conventional AFM [27], the local power behavior of the function *T*^′^(*x*) around any point 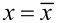 can be studied. By matching the value of *T*^′^(*x*) and its first derivative to the expression *T*′(*x*) ≈ *cx^d^*, where *c* and *d* are local constants, we obtain an expression for the term *d*, which governs the power behavior of the function *T*′(*x*), and for the term *c*

[11]
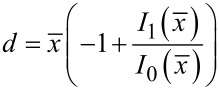


[12]
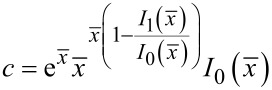


For *x* → 0, we can see that 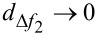, which means that 
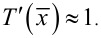
 while for larger *x*, 
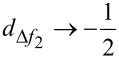
, which means that 
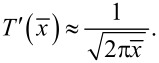
 This implies that when *A*_1_ >> 1/λ

[13]
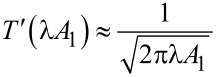


By introducing Equation 13 in Equation 9,

[14]
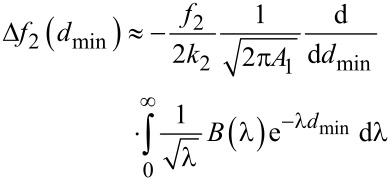


By using the property of the Laplace transform [27]

[15]
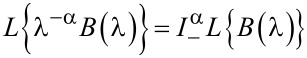


a direct relationship between Δ*f*_2_ and the half-derivative of the force 
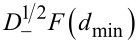
 and, alternatively, to the half-integral of the force gradient 
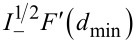
 can be found

[16]
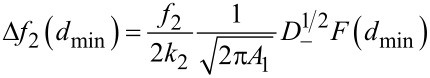


[17]
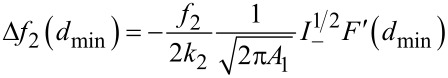


where

[18]
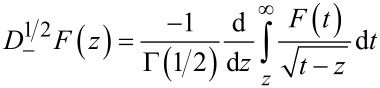


[19]
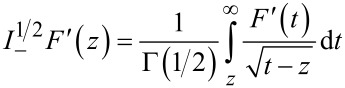


and Γ(*n*) is the Gamma function. The above fractional definitions correspond to the so-called right-sided forms of the fractional derivative and integrals [47]. Therefore the frequency shift of the second mode can be related to the half-derivative of the force, or, alternatively, it can be related to the half-integral of the force gradient whenever the amplitude of the first mode *A*_1_ is larger than the typical length scale of the interaction force. This is the typical experimental situation in bimodal FM-AFM, in which large amplitudes of the first mode are used in order to make the imaging stable [36–37] and to increase the contrast in the bimodal channel [18–19].

**Figure 2:**
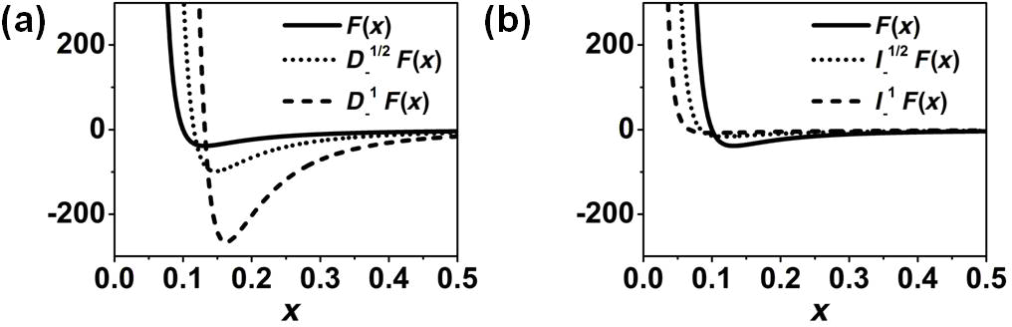
Fractional operators of (0.1^4^/*x*^6^ − 1/*x*^2^). (a) The function, half-derivative and derivative are plotted. (b) The function, half-integral and integral are plotted.

Fractional derivatives have a wide range of applications [47–48]. For example, they have been used for describing anomalous-diffusion processes, for modeling the behavior of polymers and in viscoelastic-damping models. In general, there is a near-continuous transformation of a function into its derivative by means of fractional derivatives. To illustrate this, Figure 2 shows the behavior of a function, together with its derivative, half-derivative and half-integral. We observe that the half-derivative always lies between the function and its derivative, while the half-integral is displaced to the left with respect to the function, and lies between the function and its integral.

Figure 2 shows the function (1/*x*^6^ − 1/*x*^2^), together with its derivative, its integral, its half-derivative and its half-integral. It is worth mentioning that the minimum and its *x* value for the half-derivative are situated between those of the derivative and the original function (Figure 2a). A similar situation happens with the half-integral in comparison with the function and its integral (Figure 2b).

Next, we demonstrate that the frequency shift in bimodal AFM is directly related to the half-derivative of the interaction force for two different tip–surface forces, namely Lennard-Jones forces and those described by the DMT model. We have compared the results obtained from Equation 6 with the results estimated from the half-derivative of the force (Equation 16) for a Lennard-Jones force and for the force appearing in the DMT model [49]. The force constant, resonant frequency and quality factor of the first and second flexural modes of the cantilever are, respectively, *k*_1_ = 4 N/m, *k*_2_ = 226.8 N/m, *f*_01_ = 103.784 kHz, *f*_02_ = 666.293 kHz, *Q*_1_ = 200, *Q*_2_ = 240. The ratio of the amplitudes *A*_1_/*A*_2_ = 1000 nm and the tip radius *R* = 3 nm.

The Lennard-Jones force for the interaction between two atoms is [50]

[20]
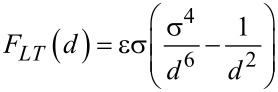


where ε is related to the depth of the energy potential and σ to the length scale of the interaction force.

For the force which appears in the DMT model [51]

[21]
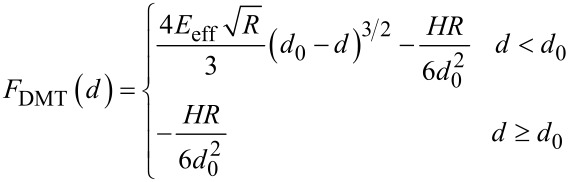


where *H* is the Hamaker constant of the long-range van der Waals forces, *d*_0_ is the equilibrium distance, *R* is the tip radius and *E*_eff_ is the effective Young’s modulus, which is related to the Young’s moduli *E_t_* and *E_s_* and Poisson coefficients ν*_t_* and ν*_s_* of the tip and sample by

[22]
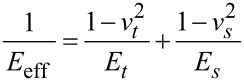


**Figure 3:**
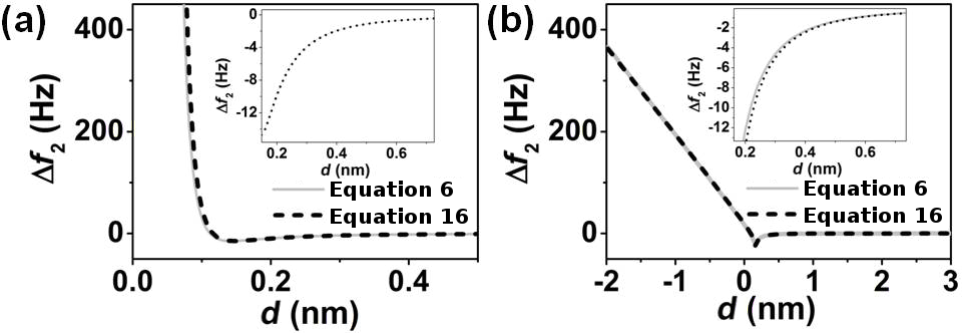
Comparison between the general expression (Equation 6) and the half-derivative (Equation 16) relationship to the frequency shift of the second mode in bimodal FM-AFM for two different forces. (a) Lennard-Jones force characterized by ε = 0.5 · 10^−20^ J and σ = 0.1 nm, and *A*_1_ = 4 nm; (b) DMT force characterized by *H* = 0.2 · 10^−20^ J, *E*_eff_ = 300 MPa, and *A*_1_ = 10 nm.

Figure 3 shows the comparison between the frequency shift of the second mode found through Equation 6 compared to that found by using the numerical half-derivative of the force (Equation 16) for a Lennard-Jones force and for a DMT force. The agreement obtained between the numerical simulations and the results deduced from the half-derivative of the interaction force are remarkable (see insets). Because the dependencies of the force on the distance in the Lennard-Jones and DTM models are rather different, we infer that the approach deduced here is general and applies to any type of force that could be found in an AFM experiment.

### Interpretation of Δ*f*_1_ in bimodal FM-AFM in terms of the half-integral of the force

For the sake of completeness, we compare the results obtained by using the expressions relating the frequency shift of the first mode and the half-integral of the force as deduced by Sader and Jarvis [27]. Δ*f*_1_ can be seen as the convolution of the force with the function 
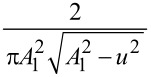
 [24]:

[23]
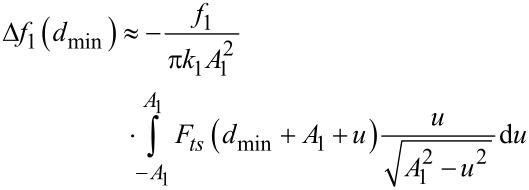


When the amplitude of the first mode is larger than the length scale of the interaction, the frequency shift of the first mode is related to the half-integral of the force:

[24]
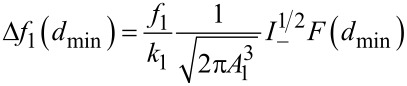


**Figure 4:**
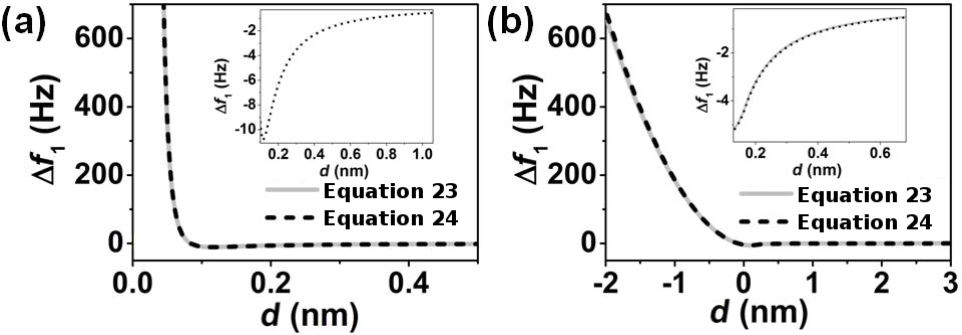
Comparison between the general expression (Equation 23) and the half-integral relationship (Equation 24) to the frequency shift of the first mode in bimodal FM-AFM for two different forces. (a) Lennard-Jones force characterized by ε = 0.5 · 10^−20^ J and σ = 0.1 nm, and *A*_1_ = 4 nm; (b) DMT force characterized by *H* = 0.2 · 10^−20^ J, *E*_eff_ = 300 MPa, and *A*_1_ = 10 nm.

Figure 4 shows the agreement obtained between the frequency shift of the first mode found through Equation 23 compared to that found by using the numerical half-integral of the force (Equation 24) for a Lennard-Jones force and for a DMT force. This agreement also supports the interpretation of the observable quantities in terms of fractional operators. In addition, it illustrates the differences of using bimodal AFM over conventional FM-AFM. When *A*_1_ is much smaller than the length scale of the interaction, the corresponding observable is proportional to the derivative both in conventional FM-AFM and in bimodal FM-AFM. However, when *A*_1_ is larger than the length scale of the interaction, Δ*f*_1_ is proportional to the half-integral of the force, while Δ*f*_2_ is proportional to the half-derivative of the force.

### Dependence of the approximate expressions for Δ*f*_1_ and Δ*f*_2_ on *A*_1_

**Figure 5:**
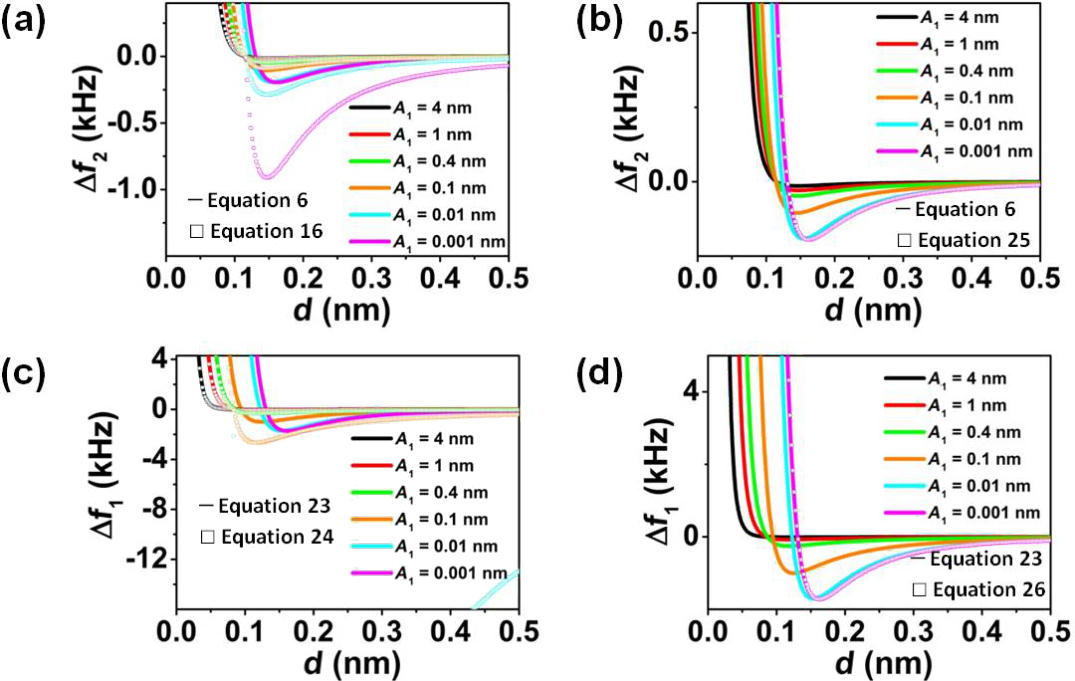
Comparison between the general expression for the frequency shift of the second mode in bimodal FM-AFM (Equation 6) and the (a) half-derivative relationship (Equation 16) and (b) derivative relationship (Equation 25). Comparison between the general expression for the frequency shift of the first mode in bimodal FM-AFM (Equation 23) and the (c) half-integral relationship (Equation 24) and (d) derivative relationship (Equation 26) for different *A*_1_ and a Lennard-Jones force characterized by ε = 0.5 · 10^−20^ J and σ = 0.1 nm, *A*_1_/*A*_2_ = 5000.

To better appreciate the differences between the frequency shifts of the first and second modes, we represent their dependence on the amplitude of the first mode (Figure 5).

When the amplitude of the first mode is much smaller than the length scale of the force, the asymptotic limit of *d*(*x*) and *c*(*x*) (Equation 11 and Equation 12) for small *x* enables us to approximate 
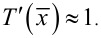
 By inserting this in Equation 9 we obtain

[25]
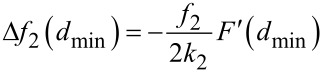


which corresponds to the experimental conditions of Naitoh et al. [35] in bimodal FM-AFM. This equation has the same dependence with the mode parameters and the force gradient as the one found for the frequency shift of the first mode in conventional FM-AFM in the limit of small amplitudes [24]

[26]
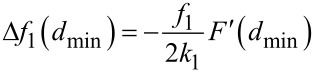


Figure 5a and Figure 5b show a comparison between the numerical results obtained from Equation 6 and the half-derivative (Equation 16) and derivative (Equation 25) for the frequency shift of the second mode approximations, which are valid in the large and small amplitude limits, respectively. For *A*_1_ above 0.1 nm, the half-derivative approximation should be used, while for *A*_1_ below 0.1 nm, the derivative approximation is a good choice. Figure 5c and Figure 5d show a comparison between the numerical results obtained from Equation 23 and the half-integral (Equation 24) and derivative (Equation 26) approximations for the frequency shift of the first mode, which are valid in the large and small amplitude limits. When *A*_1_ is above 0.4 nm, the half-integral approximation can be used, while the derivative approximation is a good choice only when *A*_1_ is smaller than 0.01 nm. There is a range between *A*_1_ = 0.01 and *A*_1_ = 0.4 nm, which depends on the typical length scale of the interaction, in which an approximation for intermediate amplitudes should be used.

## Conclusion

We have deduced an expression that relates the frequency shifts in bimodal frequency modulation AFM with the half-derivative of the tip–surface force or, alternatively, with the half-integral of the force gradient. The approximations are valid for the common experimental situation in which the amplitude of the first mode is larger than the length scale of the interaction force. The approximations are also valid for two different types of forces, namely Lennard-Jones interactions and DMT contact-mechanics forces. We conclude that the fractional-calculus approach is well suited to describe bimodal frequency modulation AFM experiments, which are characterized by the presence of several forces with different distance dependencies.
